# Distal Recurrence of Periosteal Osteosarcoma After Complete Excision of Proximal Primary Tumour With Good Excision Margins

**DOI:** 10.1080/13577140310001607310

**Published:** 2003-06

**Authors:** M. J. Barakat, C. Collins, J. H. Dixon

**Affiliations:** Southmead Hospital Redland Bristol BS6 7EX USA

## Abstract

We present this case of an unusual recurrence of a periosteal osteosarcoma in the distal right tibia 2 years after a successful proximal right tibia primary periosteal osteosarcoma excision with a successful fibular graft. This recurrence lead to a right below-knee amputation.


*Sarcoma*, June 2003, *VOL. 7, NO. 2, 79–80*

Taylor & Francis healthsciences

**CASE REPORT**

### Distal recurrence of periosteal osteosarcoma after complete excision of proximal primary tumour with good excision margins

M.J. BARAKAT, C. COLLINS & J.H. DIXON

*Southmead Hospital, Bristol, UK*

**Abstract**

We present this case of an unusual recurrence of a periosteal osteosarcoma in the distal right tibia 2 years after a successful proximal right tibia primary periosteal osteosarcoma excision with a successful fibular graft. This recurrence lead to a right below-knee amputation.

### Case report

A 34-year-old man was referred to clinic in November 1999 with a suspicious lesion in the proximal tibia. On examination, he appeared to have a non-tender fusiform bony-hard swelling of the pre-tibial areas connected to the bone proximally. A simple X-ray of the area was normal but a CT scan of his right lower limb showed the lump being mainly extraosseous, but partially intraosseous.

A biopsy of the lesion was performed, with the histological result confirming a periosteal osteosarcoma diagnosis. CT of his thorax, and a bone scan demonstrated no metastases. MRI scan of the right leg accurately confirmed the location of the lesion with no abnormality elsewhere in the tibia (Fig. 1).

Two treatment options were presented to the patient in clinic in January of 2000: (1) limb-sparing fibular graft; (2) right below-knee amputation.

He was started on chemotherapy and, in April of 2000, a decision was made for excision of the proximal perioteal osteosarcoma with fibular regrafting (resection length = 10–12 cm). A referral was made to a Consultant Plastic Surgeon, at Frenchay Hospital, and the date of 12/13 June 2000 was agreed for the operation.

The operation took place and was successful with follow-up in the joint sarcoma clinic and sequential CT scans of the thorax, which all proved to be negative for metastases. The histology report from the length of tibia resected demonstrated the presence of a periosteal osteosarcoma with incomplete (>85% overall) response to chemotherapy. The report commented that the local excision was complete with good excision margins of 1.5 cm (Fig. 2).

He represented to clinic in January 2002 with thickening of the front of the right shin just below the fibular graft site. Plain radiographic films demonstrated a radiological abnormality at that site (Fig. 3).

A biopsy of this area was performed shortly after consultation in the clinic. The histology report clearly demonstrated the recurrence of periosteal osteosarcoma in that region. The report also

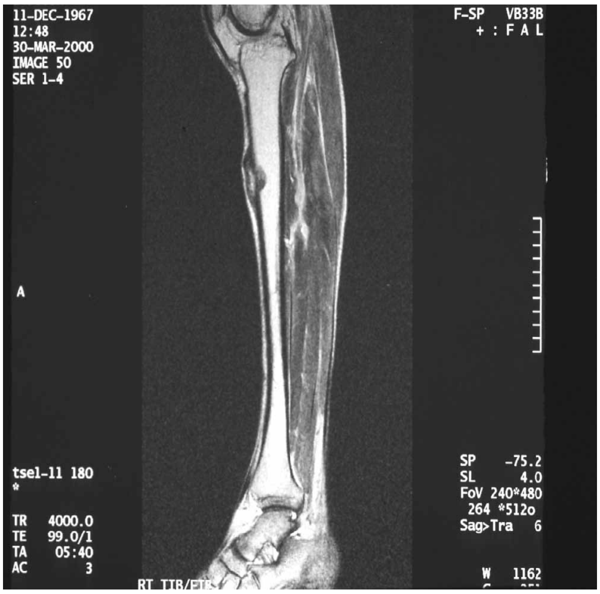

**Fig. 1.** *Periosteal osteosarcoma present at the junction of the proximal and mid third of the tibia.*

Correspondence to: M.J. Barakat, Flat 4, 13 Clarendon Rd., Redland, Bristol, BS6 7EX, UK. Tel.: 44-117-9232834; E-mail: mbarakat@blueyonder.co.uk

ISSN 1357-714X print/1369–1643 © 2003 Taylor & Francis Ltd
DOI: 10.1080/13577140310001607310

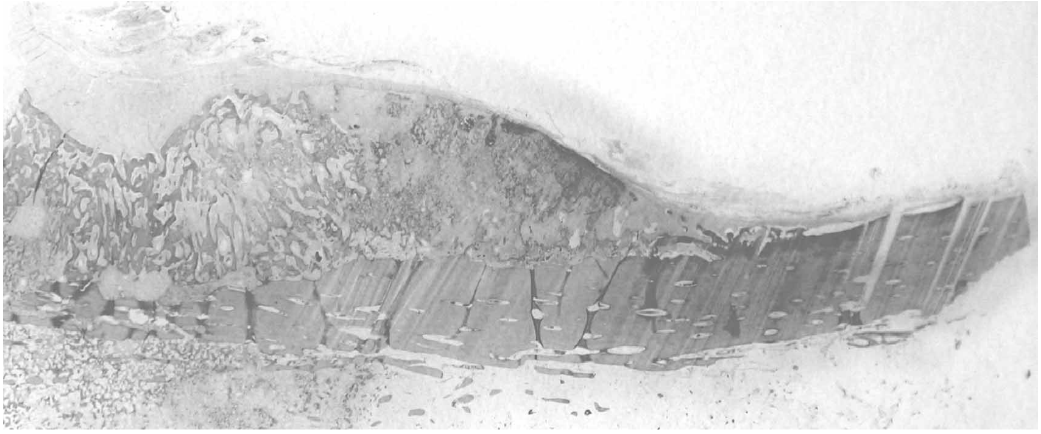

**Fig. 2.** *Histological confirmation of periosteoal osteosarcoma with good excision margin (3–5 cm).*

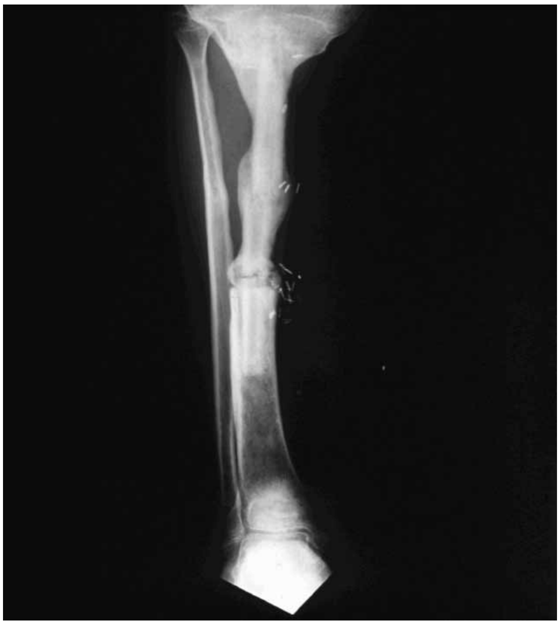

**Fig. 3.** *Radiological opacity present distal to the fibular graft.*

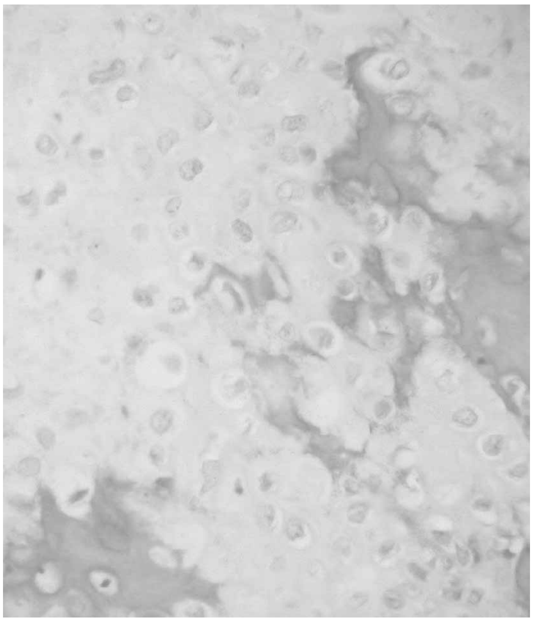

**Fig. 4.** *Histological evidence of recurrent periosteal osteosarcoma cells.*

highlighted the fact that the primary excision of the tumour demonstrated good excision margins of normal bone and periosteum (Fig. 4).

The only option left was a right below-knee amputation, which was performed on 16 January 2002. The right leg was sent for histological examination. This revealed a periosteal osteosarcoma just distal to the fibular graft with small medullary foci of high-grade osteosarcoma. There was a large tumour volume of over 100 ml. Local excision was complete. He made an uneventful recovery from this operation and went home.

### Conclusion

Although the most common site of periosteal osteosarcoma recurrence is proximal to the primary tumour, this case clearly demonstrates an example of a distal skip recurrence of a periosteal osteosarcoma. This is unique in the fact that the osteosarcoma had recurred distally, and had recurred despite the excision margins of the primary osteosarcoma excision being clear of any tumour cells histologically.[1] There has been very little research or case reports describing the details of periosteal osteosarcoma recurrence, and none describing distal skip recurrence of this tumour.

This recurrence lead to an end result of right below-knee amputation, despite the success of the fibular graft previous to the recurrence.

### Reference

1. Bacci G, Ferrari S, Longhi A, Mercuri M, Bertoni F, Gasbarrini A, Brach del Prever A, Tienghi A. Local recurrence in non-metastatic osteosarcoma of the extremities treated with neoadjuvant chemotherapy: the Rizzoli experience. Istituto Ortopedico Rizzoli, Italy, 1997.

